# Jacobs Syndrome Presenting With Delayed Puberty and Central Hypogonadism: A Rare Case Report

**DOI:** 10.1002/ccr3.73335

**Published:** 2026-08-26

**Authors:** Muhammad Hassaan Javaid, Ayesha Ahmad, Amna Ahmad, Muhammad Ajmal Khamosh, Muhammad Hanzla

**Affiliations:** ^1^ Shifa College of Medicine Islamabad Pakistan; ^2^ Khyber Medical University Peshawar Pakistan; ^3^ CMH Kharian Medical College Kharian Pakistan; ^4^ Liaquat University of Medical and Health Sciences Jamshoro Pakistan; ^5^ Sylhet MAG Osmani Medical College Sylhet Bangladesh

**Keywords:** 47,XYY syndrome, delayed puberty, hypogonadotropic hypogonadism, Jacobs syndrome, karyotype, sex chromosome aneuploidy

## Abstract

In some rare instances, patients with 47,XYY syndrome can be short in height, accompanied by hypogonadism without the characteristic tall stature. In cases where there is delayed puberty and unusual growth pattern, a complete endocrine work‐up, including GnRH and hCG stimulation tests, along with chromosome studies, is vital.

## 1 Introduction

Jacobs syndrome, which is another name for 47,XYY syndrome, is a disorder involving the number of sex chromosomes, marked by an extra copy of the Y chromosome in males, hence having the karyotype of 47,XYY. The condition occurs in about one out of every thousand male infants, thus making it among the most prevalent disorders concerning sex chromosomes; however, there is minimal diagnosis of the condition due to its varied phenotypic manifestations. Most of the patients diagnosed with the condition are detected through incidental chromosomal studies done for other reasons [1, 2].

The phenotypic characteristics of an extra Y chromosome exhibit high variability. Frequently observed phenotypes are tallness, macrocrania, muscle hypotonia, developmental delays, and neurobehavioral variations, but these traits are not necessarily seen in all patients [3]. Regarding endocrine function and fertility, the clinical profile is not fully elucidated. Although there is an older report of normal levels of testosterone and normal puberty development in the majority of cases, modern systematic studies have demonstrated the existence of patients with subclinical testicular failure, altered hormone regulation, impaired spermatogenesis, and symptoms consistent with primary gonadal insufficiency [4].

In a cross‐sectional biomarker analysis of 82 boys with 47,XYY in comparison with 66 matched controls, while the levels of total testosterone, testicular volume, and stretched penile length showed no significant difference between the two groups, boys with 47,XYY had significantly decreased inhibin B but significantly elevated anti‐Müllerian hormone (AMH) levels in contrast to controls, suggesting subtle participation of the Sertoli‐cell axis despite seemingly normal androgen production [5]. The conclusion was drawn that the endocrine phenotype of 47,XYY is heterogeneous instead of being invariably hypergonadotropic, and the current case, characterized by central hypogonadism, needs to be viewed in light of this knowledge.

The patient is a 14‐year‐old boy with Jacobs syndrome, who presented with short stature and lack of pubertal development, and was later diagnosed with central (hypogonadotropic) hypogonadism, a neuroendocrine disorder not previously reported in this particular chromosomal aberration. This case emphasizes the significance of considering chromosomal syndromes in the differential diagnosis of delayed puberty and the relevance of thorough endocrine and genetic testing in unusual cases of sex chromosome abnormalities.

## 2 Case Presentation

The patient is a 14‐year‐old boy referred due to short stature and delayed puberty. His parents indicated that he had always been short compared to his peers and had no secondary sexual development for his age. The patient denies anosmia, headaches, visual problems, or any signs of increased intracranial pressure.

Notably, the patient presented with bilateral cryptorchidism, and he had undergone bilateral orchidopexy at the age of about 2 years. There was no history of any head injury, cranial surgery, and/or irradiation to the skull. The parents were both of short stature. There was no familial history of delayed puberty, sexual disorders, endocrine disorders, and consanguinity.

The child was born after a complete gestation through a normal vaginal birth process. His prenatal history was without any problems like maternal infection, exposure to any teratogens, gestational diabetes, or hypertension. He had no history of birth asphyxia, hypoglycemia, hyperbilirubinemia, or neonatal intensive care admission. His developmental milestones were achieved appropriately, and he was exclusively breastfed as an infant.

### 2.1 Physical Examination

In terms of the physical assessment, the individual seemed to be physically fit and did not have any pain. He had normal vital signs. Anthropometric measurements revealed that the height of the individual was 141.5 cm which was below the 3rd percentile among a 14 year‐old male which was equivalent to a height SDS of −2.65 which indicated severe short stature. There was no indication of dysmorphism. No abnormality was seen in the examination of his head, neck, cardiovascular, respiratory, and abdominal systems. Hepatosplenomegaly, gynecomastia, and neurological findings were negative. His secondary sexual characteristics were in Tanner Stage I (see Table 2).

### 2.2 Investigations

The results of hormonal analysis are summarized in Table 1 below. The thyroid profile (TSH and free T4), prolactin, serum cortisol, IGF‐1, and 17‐hydroxyprogesterone were within the normal limits. Levels of LH and FSH were on the lower side of the normal limit while serum testosterone was significantly low in comparison with age‐based reference values for 14 years old male (100–600 ng/dL).

**TABLE 1:** Hormonal and biochemical investigations.

Investigation	Patient value	Reference range	Interpretation
Thyroid Stimulating Hormone (TSH)	1.82 mIU/L	1.0–7.0	Normal
Free Thyroxine (FT4)	1.13 ng/dL	0.97–1.67	Normal
Luteinizing Hormone (LH)	2.06 IU/L	1.0–3.5	Low‐normal
Follicle Stimulating Hormone (FSH)	2.64 IU/L	0–10	Low‐normal
Total Testosterone	66.1 ng/dL	100–600 ng/dL (age 14, normal puberty)	Low for age
Prolactin	5.11 ng/mL	3.0–14.7	Normal
Morning Serum Cortisol	7.29 μg/dL	4.0–19.5	Normal
Insulin‐like Growth Factor‐1 (IGF‐1)	285 ng/mL	115–498 ng/mL (age 14)	Normal (mid‐range)
Anti‐Müllerian Hormone (AMH)	42.5 ng/mL	15.0–95.0 ng/mL (Tanner Stage I)	Normal (mid‐range)
17‐Hydroxyprogesterone	0.01 ng/mL	< 2.0	Normal

Abbreviations: AMH, anti‐Müllerian hormone; FSH, follicle‐stimulating hormone; FT4, free thyroxine; IGF‐1, insulin‐like growth factor‐1; LH, luteinizing hormone; TSH, thyroid‐stimulating hormone.

The radiological evaluation revealed a bone age of 8–10 years for a patient who was chronologically 14 years old (Table 2). This is indicative of a delay of about 4–6 years. MRI of the pituitary gland was done to rule out any structural abnormality associated with hypogonadotropic hypogonadism.

**TABLE 2:** Radiological and growth assessment.

Parameter	Findings
Chronological Age	14 years
Bone Age	8–10 years
Height	141.5 cm (well below 3rd percentile for age and sex)
Height SDS	−2.65 SDS
Pubertal Stage	Tanner Stage I
Testicular Volume	3–4 mL bilaterally
Stretched Penile Length	2.5 cm

*Note:* Tanner staging based on clinical examination at the time of presentation.

Results from GnRH stimulation testing indicated a blunted response of gonadotropins, where the peak LH increased from 2.06 to 3.8 IU/L and the peak FSH increased from 2.64 to 4.1 IU/L (Table 3), which was not enough for a pubertal age individual, indicating a condition of hypogonadotropic hypogonadism and not the hypothalamo‐pituitary‐gonadal axis that was delayed. The presence of a delayed bone age, no puberty development, and abnormally normal levels of gonadotropins despite lower testosterone levels suggested isolated hypogonadotropic hypogonadism (IHH).

**TABLE 3:** GnRH stimulation test results.

Parameter	Baseline	Peak post‐GnRH	Interpretation
Luteinizing Hormone (LH)	2.06 IU/L	3.8 IU/L	Blunted peak response
Follicle Stimulating Hormone (FSH)	2.64 IU/L	4.1 IU/L	Blunted peak response

Abbreviations: hCG, human chorionic gonadotropin; LH, luteinizing hormone.

The hCG stimulation test produced a marked elevation in the serum level of testosterone (66.1–495 ng/dL), indicating that the Leydig cells retained their steroidogenic function, and suggesting that the cause was central rather than primary (Table 4).

**TABLE 4:** hCG stimulation test results.

Parameter	Baseline	Post‐hCG	Interpretation
Serum Testosterone (ng/dL)	66.1	495	Adequate Leydig cell response
Luteinizing Hormone (IU/L)	2.06	—	Central hypogonadism suggested
Testicular Volume	3–4 mL	No significant change	Prepubertal testes

Differentiating IHH from constitutional delay of growth and puberty (CDGP) at 14 years of age and Tanner I stage of development poses a challenging diagnostic problem as basal levels of gonadotropins and testosterone levels may be similar in the two conditions. The following characteristics made the diagnosis of a central lesion more likely in this case: (1) the suboptimal gonadotropin response to GnRH stimulation as noted above, which is not seen in CDGP, in which the hypothalamic–pituitary–gonadal axis functions normally but is just delayed in activation; (2) a marked increase of testosterone after hCG stimulation, indicating Leydig cells' function, which indicates a lesion in the hypothalamo–hypophyseal region and not in the gonad; (3) bone age lag consistent but not entirely explaining the degree of sexual immaturity; and (4) lack of family history of pubertal delay, a characteristic of CDGP. We acknowledge that longitudinal follow‐up through and beyond expected puberty remains the definitive discriminator between IHH and CDGP, and this is noted as a limitation of the case.

### 2.3 Management

Due to the high degree of psychosocial disturbance from the lack of development of sexual characteristics and short stature, androgen therapy was commenced. A transdermal patch was chosen as an alternative to the injectable form of testosterone for gradual induction of puberty, taking into account growth rate and bone maturity, since early closure of the epiphyses is a definite risk in light of the subject's poor stature status. The presence of a 47,XYY karyotype was thoroughly explained to the subject and his family.

Chromosomal analysis of peripheral blood chromosomes showed the presence of 47,XYY karyotype non‐mosaic in all 20 cells examined, excluding mosaicism such as 45,X/47,XYY or 46,XY/47,XXX that sometimes may be seen in patients with growth retardation or mixed gonadal dysgenesis. The diagnosis, difference from Klinefelter's syndrome, and management, including expected central and not chromosomal origin of his hypogonadism, good prognosis of successful puberty induction using androgen treatment and necessity of follow‐up of fertility issues were discussed with the patient and his parents.

### 2.4 Follow‐Up

During the six‐month follow‐up, there was an increase in virilization in terms of penile enlargement and sparse pubic hair growth, and the patient had grown 11 cm in height. Puberty staging had reached early Tanner Stage II. There was no enlargement of the testes, indicating that puberty was still incomplete, which was expected based on the central cause of hypogonadism, since the administration of exogenous androgens leads to peripheral virilization but not to seminiferous tubular growth, which needs follicle‐stimulating hormone (FSH) stimulation (Table 4). Continued monitoring of the patient's endocrinology was scheduled (Table 5).

**TABLE 5:** Clinical response at six‐month follow‐up on topical testosterone.

Parameter	Baseline	6‐month follow‐up
Stretched Penile Length	2.5 cm	Minimal increase
Penile Girth	Reduced	Noticeable increase
Pubic Hair	Absent	Sparse
Axillary Hair	Absent	Absent
Tanner Stage	I	Early II
Height Gain	—	+11 cm

*Note:* Tanner staging based on clinical examination.

## 3 Discussion

The typical presentation of Jacobs syndrome is with tall stature, macrocephaly, and a wide variety of neurological manifestations; the development of puberty has been reported to be normal or minimally impacted [3, 4]. However, our patient's presentation is an unusual case that contrasts with what is expected: he was a short statured individual instead of a tall statured one, and he had hypogonadotropic hypogonadism.

This seeming discrepancy should be explained with consideration of the latest information on biomarkers indicating heterogeneity of the endocrine phenotype in 47,XYY syndrome, which has been proven to be more varied than suggested by the traditional description of “tall stature, hypergonadotropic hypogonadism”. Specifically, in the biggest pediatric biomarker series performed so far, patients with XYY did not show any difference between them and control participants in terms of testosterone, testicular volume and penile length; however, their level of inhibin B was significantly decreased and their AMH was increased—the indication of the impaired testicular function that does not result in clear deficit of androgens in most patients [5]. Our patient's markedly reduced height (SDS −2.65) and central hypogonadism sit outside even this expanded phenotypic range, underscoring that individual 47,XYY presentations can diverge substantially from both the classical description and the emerging heterogeneous picture.

Short stature in the present case can be explained by the following reasons: significant delay in skeletal development (bone age 8–10 years while chronological age is 14 years); presence of a genetic predisposition to short stature in the family (parents have short stature); failure to go through the sex hormone‐induced pubertal growth spurt owing to hypogonadotropic hypogonadism [6, 7].

However, most of the reproductive abnormalities described in individuals with 47,XYY syndrome are hypergonadotropic hypogonadism, which is characterized by primary failure of the testes and presents phenotypically similar to Klinefelter's syndrome, albeit with less severe symptoms [8]. Unlike our case, the patient exhibited signs of hypogonadotropic hypogonadism, characterized by subnormal levels of testosterone with abnormally low‐normal levels of LH and FSH, implying that there was impairment of the hypothalamic–pituitary axis and not the gonads [9]. A significant increase in testosterone levels from 66.1 to 495 ng/dL, post‐stimulation test, confirmed the presence of active Leydig cells for steroidogenesis; thus, the site of malfunction was within the HPG axis.

It is unclear whether this constitutes an incidental association between the two separate anomalies of 47,XYY and idiopathic hypogonadotropic hypogonadism or a rare neurological presentation of the chromosomal anomaly itself. The presence of mutations in candidate genes responsible for congenital hypogonadotropic hypogonadism, including KAL1, FGFR1, or GNRHR, associated with or perhaps regulated by the aneuploidy may be considered in future studies [10, 11].

Another clinical finding worth mentioning is that of bilateral cryptorchidism treated surgically with orchidopexy at the age of 2 years. The incidence of cryptorchidism is more common in patients with sex chromosome aneuploidy when compared to the general population [12]. Although the presence of cryptorchidism suggests an increased risk of primary injury to the testis due to the long‐term heat exposure of the germinal epithelium, the fact that the patient has normal AMH levels and normal stimulation of testosterone production by hCG indicates that there is no primary impairment of spermatogenesis [13].

Jacobs syndrome tends to be overlooked because of its broad and usually subtle clinical presentation [1]. In this case, the delay in puberty was what sparked the process resulting in the karyotyping. Individualized management is essential: androgens are required to ensure virilization, increase bone mineral density, and improve psychosocial development, yet they have to be used cautiously in individuals with reduced height growth capability to prevent early epiphyseal closure [13]. The use of topical testosterone led to a slow and natural increase in hormonal levels, leading to an 11‐cm growth spurt and virilization to stage II of Tanner's staging system after 6 months. The lack of increase in testicular size reflects the central cause of hypogonadism, which indicates that while the administration of testosterone leads to peripheral virilization, it does not induce spermatogenesis, which is under the control of follicle‐stimulating hormone (FSH) [14]. Future management strategies might include pulsatile GnRH therapy or combined FSH/LH therapy.

One problem with this report is that, as is the case with any individual patient, a follow‐up examination after puberty is necessary in order to be able to differentiate between central hypogonadotropic hypogonadism and a particularly severe constitutional delay in this particular patient.

## 4 Conclusion

This case demonstrates that the manifestation of 47,XYY syndrome may be more varied than previously thought and may include short stature and central hypogonadism, which are not common findings in patients with 47,XYY syndrome. Physicians treating adolescents with pubertal delay and abnormal endocrine parameters must be vigilant in performing chromosome studies even in the absence of typical clinical manifestations, like tall stature. Endocrine work‐up, particularly assessing the function of both the central and peripheral gonads through GnRH and hCG stimulation tests, is necessary to determine the underlying cause of hypogonadism and provide proper treatment.

## Author Contributions

**Amna Ahmad:** formal analysis, investigation, validation. **Ayesha Ahmad:** validation, software, data curation, visualization. **Muhammad Hassaan Javaid:** conceptualization, methodology, software, formal analysis, project administration. **Muhammad Hanzla:** writing – review and editing. **Muhammad Ajmal Khamosh:** supervision, formal analysis, investigation, project administration, resources.

## Funding

The authors have nothing to report.

## Ethics Statement

Ethical Approval is not required for case reports at our institution.

## Consent

Written informed consent was obtained from the patient's legal guardian for the publication of this case report and any accompanying clinical data. Written informed consent for publication was obtained from the patient's guardian. A copy of the consent form is available for review by the Editor‐in‐Chief of this journal upon request.

## Conflicts of Interest

The authors declare no conflicts of interest.

## Data Availability

Data sharing not applicable as no new datasets were generated or analyzed in this case report.
